# Oral esketamine for treatment-resistant depression: rationale and design of a randomized controlled trial

**DOI:** 10.1186/s12888-019-2359-1

**Published:** 2019-11-29

**Authors:** Sanne Y. Smith-Apeldoorn, Jolien K. E. Veraart, Jeanine Kamphuis, Antoinette D. I. van Asselt, Daan J. Touw, Marije aan het Rot, Robert A. Schoevers

**Affiliations:** 1 Department of Psychiatry, University of Groningen, University Medical Center Groningen, PO box 30.0001, 9700 RB Groningen, The Netherlands; 2 Department of Psychiatry, PsyQ Haaglanden, Parnassia Psychiatric Institute, The Hague, The Netherlands; 3 Department of Epidemiology, University of Groningen, University Medical Center Groningen, Groningen, The Netherlands; 4 Department of Clinical Pharmacy and Pharmacology, University of Groningen, University Medical Center Groningen, Groningen, The Netherlands; 5 Department of Psychology, University of Groningen, Groningen, The Netherlands

**Keywords:** Esketamine, Oral administration, Clinical trial, Treatment-resistant depression

## Abstract

**Background:**

There is an urgent need to develop additional treatment strategies for patients with treatment-resistant depression (TRD). The rapid but short-lived antidepressant effects of intravenous (IV) ketamine as a racemic mixture have been shown repeatedly in this population, but there is still a paucity of data on the efficacy and safety of (a) different routes of administration, and (b) ketamine’s enantiomers esketamine and arketamine. Given practical advantages of oral over IV administration and pharmacodynamic arguments for better antidepressant efficacy of esketamine over arketamine, we designed a study to investigate repeated administration of oral esketamine in patients with TRD.

**Methods:**

This study features a triple-blind randomized placebo-controlled trial (RCT) comparing daily oral esketamine versus placebo as add-on to regular antidepressant medications for a period of 6 weeks, succeeded by a follow-up of 4 weeks. The methods support examination of the efficacy, safety, tolerability, mechanisms of action, and economic impact of oral esketamine in patients with TRD.

**Discussion:**

This is the first RCT investigating repeated oral esketamine administration in patients with TRD. If shown to be effective and tolerated, oral esketamine administration poses important advantages over IV administration.

**Trial registration:**

Dutch Trial Register, NTR6161. Registered 21 October 2016.

## Background

Major depressive disorder (MDD) is one of the most impactful medical conditions worldwide in terms of individual suffering, loss of productivity, and health care costs [1, 2]. Unfortunately, current treatments for depression fail to achieve remission in approximately 30% of patients [3]. This is known as treatment-resistant depression (TRD).

TRD contributes disproportionately to the disease burden of MDD. This burden exponentially increases the longer TRD persists, with increasing risk of impaired functional and social functioning [4], vast losses in quality of life for both patients and people close to them [4, 5], and increasing risk of somatic morbidity [6, 7]. Moreover, TRD is associated with an impressive financial burden to society, due to patients’ more extensive and costly use of medical services, as well as to their loss of productivity [4, 5, 8]. Hence, there is an urgent need to develop more efficacious therapeutic strategies for MDD generally, and for TRD specifically.

It has been two decades since a single intravenous (IV) administration of the anaesthetic agent ketamine was first reported to have antidepressant effects in patients with MDD [9]. Since then, accumulating data have confirmed ketamine’s antidepressant effects [10, 11]. Two features of these data are most striking. Firstly, response can become manifest within hours after administration. Secondly, this response takes place even in patients with TRD.

In most patients, the therapeutic effects of a single IV administration of ketamine last about 1 week [11, 12]. These effects can be extended with repeated IV administration [13–15]. However, this procedure is invasive, costly, and often brings about acute psychiatric (e.g., dissociation, anxiety, agitation) and somatic (e.g., headache, dizziness, cardiovascular) side effects [16]. These disadvantages present major obstacles to clinical applicability, especially in community settings.

To date, several uncontrolled studies (reviewed by Schoevers et al. [17] and Rosenblat et al. [18]) and three small controlled studies [19–21] have reported on the antidepressant properties of oral ketamine. They suggest that oral ketamine may also be effective in patients with TRD, and that side effects are overall more acceptable compared to IV administration. Besides, data from chronic pain management indicate that oral ketamine can often be safely used for longer periods of time, including at home [17]. Thus, oral ketamine may be a suitable alternative for IV ketamine in the treatment of TRD. However, the literature on oral ketamine is scarce, and the bioavailability of oral ketamine has been little studied. There remains a need to conduct larger controlled studies, and to examine the pharmacokinetics and pharmacodynamics of oral ketamine [22].

In most TRD studies conducted to date, ketamine has been administered as a racemic mixture comprised of the R-(−) enantiomer of ketamine (arketamine) and the S-(+) enantiomer (esketamine). In the brain, ketamine modulates glutamate transmission by acting as an N-methyl-D-aspartic acid (NMDA) receptor antagonist. The NMDA receptor binding affinity of esketamine is three to four times higher than that of arketamine [23, 24]. As the majority of ketamine’s antidepressant properties are believed to stem from its impact on glutamate neurotransmission, theoretically esketamine might yield the best therapeutic effect. Indeed, rapid and robust antidepressant effects of esketamine have been observed in patients with TRD [25–27]. Besides, compared to racemic ketamine and arketamine, esketamine is believed to have fewer side effects [26, 28, 29]. To date there have been no controlled oral esketamine studies.

Ketamine also has other effects that may be linked to its antidepressant properties. It is used for the treatment of chronic pain [17] and treatment-resistant anxiety disorders [30], conditions that are often comorbid with MDD [31, 32]. Besides, ketamine could play a role in smoking cessation, as the pharmacodynamic effects of nicotine may involve NMDA receptors [33].

In summary, given (1) the advantages of oral over IV administration, and (2) pharmacodynamic arguments for a better antidepressant efficacy of esketamine over racemic ketamine and arketamine, oral esketamine is a promising addition to our currently available treatment armamentarium for depression. To investigate repeated administration of oral esketamine in patients with TRD, we designed a triple-blind randomized controlled trial (RCT).

The primary aim of this RCT is to examine the antidepressant properties of oral esketamine in patients with TRD, as determined using clinician rating scales. Secondary aims involve the effects of oral esketamine on self-reported severity of depression, depressive symptom dimensions, hedonic capacity, suicidal ideation, cognitive functioning, quality of life, safety, tolerability, and its effects in specific subgroups of patients. Apart from these aims, we will address additional relevant questions regarding (1) therapeutic effects of oral esketamine on pain, anxiety and nicotine addiction, (2) its bioavailability and mechanism of action, and (3) its economic impact.

## Methods

### Study design

This study features a triple-blind RCT with two parallel arms, as add-on to regular antidepressant medication: an esketamine (intervention) group and a placebo (control) group. The study has a total duration of 10 weeks: 6 weeks of study medication and 4 weeks of follow-up. All patients who complete the trial are subsequently offered an off-label esketamine treatment extension. This extension will be described in more detail elsewhere.

### Study management

This study is approved by the Medical Ethics Review Committee of the University Medical Center Groningen (UMCG) in the Netherlands (file number M16.198879) and registered at the Dutch Trial Register (trial number NTR6161). The independent Clinical Research Office (CRO) of the UMCG and an independent Data Safety and Monitoring Board (DSMB) oversee the conduct of the study. The CRO executes an audit of the trial system twice a year. The DSMB meets every 6 months to discuss study progress and patient safety and provide feedback to the investigators.

The study is conducted at three centers in the Netherlands: University Center of Psychiatry in Groningen, Pro Persona Depression Expertise Center in Nijmegen, and Parnassia Psychiatric Institute in The Hague.

### Study treatment

Patients randomized to the intervention arm take capsules containing oral esketamine three times a day during 42 consecutive days. During the first 3 days, dosages are gradually increased from 10 mg at administration 1 (day 1) to 30 mg at administration 9 (day 3). During the last 3 days, dosages are gradually decreased from 30 mg at administration 118 (day 40) to 10 mg at administration 126 (day 42). Patients randomized to the control arm take placebo capsules containing microcrystalline cellulose and magnesium stearate. Treatment compliance is assessed during every visit.

### Sample

#### Recruitment

Psychiatry departments and patient and family associations throughout the Netherlands are involved in recruitment, and advertisement takes place by various media. Prior to screening, potential participants receive an oral and written explanation of study procedures, potential benefits, and potential risks. They are informed that participation is voluntary and that they are free to withdraw at any time for any reason. Before enrolment, written informed consent is obtained from each patient.

#### Eligibility

Patients are selected for study enrolment based on the inclusion and exclusion criteria listed in Table 1. During the study, investigators can decide to withdraw a participant for urgent medical reasons, or if the situation of a participant changes such that he or she is no longer eligible to participate.

**Table 1:** Inclusion and exclusion criteria

Subject eligibility	
Inclusion criteria	1. Age 18 to 80 years; 2. Current major depressive episode according to the DSM-5, ascertained by the MINI; 3. Depression at least moderately severe, defined by a score > 18 on the HDRS_17_; 4. TRD, defined as insufficient lifetime response to 3 or more different classes of antidepressant drugs, given for at least 4 weeks and in an adequate dose; 5. Stable dose of current antidepressant drug for at least 4 weeks prior to study initiation; 6. Good understanding of spoken and written Dutch.
Exclusion criteria	1. Meet DSM-5 criteria for current major depressive episode with psychotic features, bipolar disorder, past or current psychotic disorder, past or current moderate or severe substance dependence, or personality disorder as a primary diagnosis; 2. Recent (within the last 4 weeks) or current use of non-prescribed psychoactive compounds; 3. Recent (within the last 4 weeks) or current use of benzodiazepines in excess of 2 mg lorazepam or equivalent per day; 4. Current electroshock therapy; 5. Active suicidal intent, defined by a score > 2 on item 3 of the HDRS_17_; 6. Pregnancy or lactation; 7. A relevant somatic disorder, ascertained by physical examination, electrocardiogram, and blood tests; 8. Use of medication that ketamine interacts with on a major level according to the Drug Interactions Checker [34], including monoamine oxidase inhibitors.

*DSM-5* 5th edition of the Diagnostic and Statistical Manual of Mental Disorders [35], *HDRS*_17_ 17-item Hamilton Depression Rating Scale [36, 37], *MINI* Mini International Neuropsychiatry Interview [38], *TRD* Treatment-resistant depression

#### Statistical power

At the time of sample size calculation, one open-label study had shown antidepressant effects of oral racemic ketamine in 57% of patients [39]. Previously, another open-label study had shown antidepressant effects of oral esketamine in 50% of patients [40]. This indicates a response rate of oral (es)ketamine of 50–57%. As the lack of a control group in these studies might have inflated response rates, in the present trial a response rate of 40% was estimated for the intervention group. For the control group, a response rate of 15% was estimated. This was based on previous studies showing a placebo response in 14.4% of TRD patients [41].

To detect a significant difference in response rate between groups, with the two-sided significance level set at 95% (α = 0.05) and a power of 0.8, 57 participants per group should complete the trial. Assuming a 10% drop-out rate, 64 participants will be included in both groups, leading to a total of 128 participants.

### Randomization and blinding

Participants are randomly allocated in a 1:1 manner to either treatment group. Randomization is conducted through ALEA Clinical web application. Blinding takes place at the level of participants, clinicians, and study staff. Placebo capsules are matched to esketamine capsules in shape, smell, and colour. All capsules are sealed in identical blisters. Blisters are labelled as trial medication, and given a trial number by the manufacturer ACE Pharmaceuticals. A list with trial medication numbers and the corresponding allocated treatment is stored at the Department of Clinical Pharmacy and Pharmacology of the UMCG. None of the study team members have access to the list until the trial is finished, or unless something unexpected happens that warrants breaking the blind. The success of blinding is tested by asking participants and data collectors at the end of the intervention period which group they thought participants were in, and by comparing these data with the allocation data after unblinding.

### Tests and measures

#### Testing procedure

**Fig. 1:**
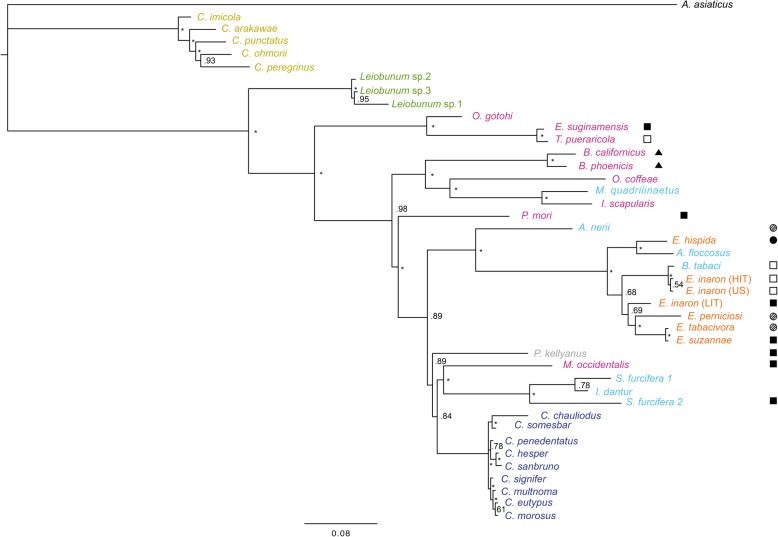
Trial flowchart. Schematic overview of the study design. T: Number illustrates number of weeks after baseline

All participants are measured before (at baseline), during (after 1, 2 and 4 weeks), and at the end (after 6 weeks) of treatment. Additionally, to determine how long therapeutic effects can be retained, follow-up assessments are planned after 1 (week 7), 2 (week 8) and 4 (week 10) weeks. All data are entered electronically. Original study forms are stored in a secure and accessible place and manner. Figure 1 represents the research procedure schematically.

#### Primary outcomes

In line with the primary aim of the study, antidepressant efficacy is measured by 1) response, defined as ≥50% decrease in total 17-item Hamilton Depression Rating Scale (HDRS_17_) score between pre-treatment and end-of-treatment; 2) partial response, defined as 25–49% decrease in total HDRS_17_ score between pre-treatment and end-of-treatment; 3) change in depression symptom severity, expressed as a change in total HDRS_17_ score between pre-treatment and end-of-treatment. The HDRS_17_ is a 17-item clinician-rated semi-structured interview [36, 37], that is used to assess the severity of depressive symptoms. The HDRS_17_ is completed only by trained clinicians and researchers. Inter-rater reliability is determined twice a year: an intraclass correlation coefficient of > 0.50 (at least moderate agreement) is pursued [42].

#### Secondary outcomes

The Inventory of Depressive Symptomatology (IDS-SR) is a 30-item self-report questionnaire that is used to assess the severity of depressive symptoms as reported by the patient [43]. The Clinical Global Impression (CGI) is a 2-item clinician-rated instrument that is used to assess the overall depression severity (CGI-severity), and the general effect of therapy on the overall depression severity (CGI-improvement) [44]. Hedonic capacity is assessed by the Snaith Hamilton Anhedonia and Pleasure Scale (SHAPS), a 14-item self-report questionnaire [45]. The SHAPS assesses hedonic capacity separately from other depressive symptoms, as anhedonia represents a central construct in MDD with multiple aspects, that is often undervalued in current MDD severity measurements [46]. The Beck Scale for Suicide Ideation (BSS) is a 21-item self-report questionnaire that is used to assess the severity of suicidal ideation [47] – separately from other depressive symptoms, as ketamine might reduce suicidal ideation partly independent from its effect on MDD in general [48, 49]. Cognitive functioning is measured by the Autobiographical Memory Test (AMT), involving the presentation of 10 cue words varying in emotional valence. Participants are asked to respond to each cue with a *specific* event that the cue reminds them of [50]. Health related quality of life is assessed by the 5-level version of the EuroQol 5D (EQ-5D-5 L), a self-report questionnaire comprising 5 dimensions (mobility, self-care, usual activities, pain/discomfort, and anxiety/depression), complemented with a visual analogue scale representing general health [51].

Outcomes of adverse events and side effects include the Questionnaire for Psychotic Experiences (QPE) [52], Dissociation Tension Scale (DSS) [53], Iowa Sleep Disturbance Inventory (ISDI) [54], and Systematic Assessment for Treatment Emergent Events (SAFTEE) [55]. Safety and tolerability will also be evaluated via heart rate, blood pressure, weight, and liver enzyme levels testing.

Outcomes that will be used to identify predictors that distinguish patients who can benefit from treatment with oral esketamine include: demographics, the Dutch Measure for quantification of Treatment Resistance in Depression (DM-TRD) [56], the NEO Five-Factor Inventory (NEO-FFI) [57] neuroticism subscale, and the credibility/expectancy questionnaire (CEQ) [58].

#### Additional outcomes

Pain is measured by the Graded Chronic Pain Scale (GCPS) [59], anxiety by the Beck Anxiety Inventory (BAI) [60], and nicotine dependence by the Fagerström Test for Nicotine Dependence (FTND) [61].

We will explore the pharmacokinetics of oral esketamine and its main metabolite esnorketamine, and the genotype of the Cytochrome P450 (CYP) enzymes involved in the metabolism. We will also describe the effects of esketamine on biomarker patterns and gene expression patterns that are related to the pathophysiology of depression [62].

Economic evaluation of treatment with oral esketamine as compared to placebo will be conducted from a societal perspective. A budget impact analysis (BIA) will be conducted to inform decision-makers about the financial consequences of the adoption and diffusion of treatment with oral esketamine for TRD in the Dutch healthcare system.

All measures and associated assessment time points are shown in Table 2.

**Table 2:** Schedule of assessments

Measurement instruments	Assessment target	Time points^a^							
		Baseline	Intervention period				Follow-up		
		< T0	T1	T2	T4	T6	T7	T8	T10
Primary and secondary outcomes									
HDRS_17_	Depressive symptoms (clinician-rated)	x		x	x	x	x	x	x
IDS-SR	Depressive symptoms (patient-rated)	x		x	x	x	x	x	x
CGI	Overall depression severity and change	x		x	x	x	x	x	x
SHAPS	Hedonic capacity	x				x			x
BSS	Suicidal ideation	x				x			x
AMT	Autobiographical memory	x				x			x
EQ-5D-5 L	Health related quality of life	x				x			x
QPE	Psychotic experiences	x	x			x			x
DSS	Dissociative features	x	x			x			x
ISDI	Sleep disturbance	x	x			x			x
SAFTEE	Side effects in any organ system	x	x	x	x	x	x	x	x
Physical examination	Heart rate, blood pressure, weight	x	x	x	x	x	x	x	x
Blood collection (I)	Liver enzyme levels	x				x			
Demographics questionnaire	Demographics	x							
DM-TRD	Treatment-resistance	x							
NEO-FFI	Neuroticism	x							
CEQ	Credibility and expectancy of intervention	x							
Additional outcomes									
BAI	Anxiety symptoms	x				x			x
GCPS	Pain	x				x			x
FTND	Nicotine dependence	x				x			x
Blood collection (II)	Biomarkers, gene expression, pharmacokinetics, CYP enzymes	x	x			x			x
Urine collection	Biomarkers	x				x			x
ZGV	Health care consumption	x				x			x

^a^Number illustrates number of weeks after baseline. *AMT* Autobiographical Memory Test, *BAI* Beck Anxiety Inventory, *BSS* Beck Scale for Suicide Ideation, *CEQ* Credibility/expectancy questionnaire, *CGI* Clinical Global Impression, *CYP* Cytochrome P450, *DM-TRD* Dutch Measure for quantification of Treatment Resistance in Depression, *DSS* Dissociation Tension Scale, *EQ-5D-5 L* EuroQol 5D, *FTND* Fagerström Test for Nicotine Dependence, *GCPS* Graded Chronic Pain Scale, *HDRS*_*17*_ Hamilton Depression Rating Scale, *IDS-SR* Inventory of Depressive Symptomatology, *ISDI* Iowa Sleep Disturbance Inventory, *NEO-FFI* NEO Five-Factor Inventory, *QPE* Questionnaire for Psychotic Experiences, *SAFTEE* Systematic Assessment for Treatment Emergent Events, *SHAPS* Snaith Hamilton Anhedonia and Pleasure Scale, *ZGV* Health care use questionnaire (Zorggebruik Vragenlijst) – adapted from the TicP [63] to the context of the current study

### Statistical analysis plan

The efficacy and safety of esketamine will be tested by the use of intention-to-treat and per-protocol linear and logistic mixed models. The effects on biomarker patterns will be tested using Receiver Operating Characteristics (ROC) analyses in combination with phenotype randomization. Pharmacokinetic models will be built by using population pharmacokinetic modelling software (MWPharm) using Iterative-2-stage Bayesian techniques, and will include the absorption (esketamine) or formation (esnorketamine) constant, bioavailability, volume of distribution (relative to bioavailability), and total body clearance (relative to bioavailability). Next, these models will be used to make an estimation of exposure. These data will be analysed using descriptive statistics. The relationship between exposure variables, efficacy and safety will be explored by using regression models and box-and-whisker plots.

EQ-5D-5 L data will be converted into Quality Adjusted Life Years (QALYs) using the Dutch tariffs [64]. Healthcare resource use, loss of productivity, and informal care will be recalculated into societal costs according to the Dutch guidelines for economic evaluation in healthcare [65]. Cost-effectiveness and cost-utility will be reported as incremental costs per point gained on the HDRS_17_ and per QALY gained, respectively. Uncertainty surrounding the outcomes will be assessed by bootstrap analyses and cost-effectiveness acceptability curves.

## Discussion

The current RCT examines the effects of repeated administration of oral esketamine as add-on to regular antidepressant medication in patients with TRD. As such, the study addresses the urgent need to identify improved treatment strategies for patients with TRD. The rapid antidepressant effects of IV ketamine have been repeatedly shown in this population, but these effects are mostly transient and the IV administration has disadvantages.

Several study design considerations merit further discussion. Firstly, our trial involves oral rather than IV administration of ketamine. If proven to be effective, oral ketamine poses important advantages over IV ketamine. As previously mentioned, IV administration is costly and impractical. Moreover, it is inconvenient for patients, and associated with more side effects than other routes of administration. This limits the practical utility of IV ketamine in real-world settings.

Compared to IV ketamine, oral ketamine has a variable and low bioavailability of 17–23% [66, 67]. The absorption rate of oral ketamine appears to vary substantially, both between and within patients, possibly due to variation in gut motility, the state of the stomach, food intake, and genetic factors [68]. Additionally, oral ketamine undergoes extensive first-pass metabolism, which is influenced by individual differences in cytochrome phenotypes. While a low bioavailability may negatively influence the efficacy of oral ketamine, the extensive first-pass effects might also have a positive consequence. Namely, norketamine – ketamine’s main metabolite – concentrations are relative high after oral administration of ketamine [68]. In rodent models, norketamine’s antidepressant effects appear to be similar to those of ketamine, but they are associated with less behavioural and biochemical abnormalities [69]. These findings suggest that norketamine might serve as an alternative to ketamine. In our oral (es)ketamine study, we assume that relatively high norketamine levels will be reached during the steady-state phase. Patients might subsequently report similar antidepressant effects with relatively few side effects.

While some oral (es)ketamine studies have shown an antidepressant effect within hours after administration, most have shown this only after weeks of treatment [18]. In general a more rapid onset of action with IV rather than oral administration of antidepressant medication is not uncommon, understandable from a pharmacological perspective, and not associated with increased efficacy [70]. The treatment duration of 6 weeks in our study was set to be long enough to detect even a delayed antidepressant effect. Besides, a longer treatment duration might enhance the duration of the response to ketamine, and therefore provide patients a better opportunity to recover. Previous research indeed suggests a prolonged response duration after repeated compared to single-dose ketamine administration (e.g. [13–15, 25]).

Some studies have explored other strategies to extend the antidepressant effect of a single ketamine dose, for example by means of lithium, riluzole, or cognitive behavioural therapy [71–73]. Continuation with regular antidepressant medication, as required in this study, might also contribute to relapse prevention, as is seen in studies on relapse prevention after index electroconvulsive therapy for TRD [74]. Ketamine has been added to treatment as usual in previous studies [20, 25]. This is considered safe, as ketamine has no major interactions with regular antidepressant medications [75].

Both oral and intranasal administration of ketamine could be suitable alternatives to IV administration, as they both improve applicability and offer the possibility of self-administration. Advantages of oral administration over intranasal administration are that the oral route is associated with the lowest abuse liability [76] and seems the most practical [22]. In March 2019 the US Food and Drug Administration approved an esketamine nasal spray, developed by Janssen Pharmaceutical Companies of Johnson & Johnson, for the treatment of TRD. However, as the spray will only be available via a restricted distribution system, its accessibility might remain limited [22]. Furthermore, the costs per patient per month that have been communicated are very substantial [77]. It is therefore still necessary to consider alternative administration routes.

A second study design consideration that merits further discussion is that our trial involves esketamine rather than racemic ketamine. In the Netherlands, as in some other European countries, only esketamine is available for medical use [78]. As mentioned earlier, compared to racemic and arketamine, esketamine shows a higher affinity for the NMDA receptor and might be associated with fewer side effects. Esketamine might therefore be a more potent and safer antidepressant. However, which ketamine form is preferential remains an important research question. We expect to contribute to this field with the study presented here. Also of note, while no clinical study to date has directly compared the antidepressant properties of the two enantiomers directly or with the racemic mixture, the first IV trial is currently being conducted [79].

We derived the daily esketamine dose used in our study from previous studies on (es)ketamine, including our pilot study (Smith-Apeldoorn SY, Veraart JKE, Ruhé HG, Aan het Rot M, De Boer MK, Schoevers RA. Oral S-ketamine for treating treatment-resistant depression - a case series. In preparation). Initially, the daily dose was based on the most commonly studied IV dose of 0.5 mg/kg racemic ketamine, i.e. 0.25 mg/kg esketamine. If 0.25 mg/kg esketamine accounts for 80% of the NMDA receptor antagonism and 0.25 mg/kg arketamine accounts for the remaining 20%, then about 0.3 mg/kg esketamine counts for similar NMDA receptor antagonism as 0.5 mg/kg racemic ketamine. Assuming a 20% bioavailability, a single dose of 1.5 mg/kg oral esketamine should then equal a single dose of 0.5 mg/kg IV racemic ketamine in NMDA receptor antagonism. However, because of the repeated administration and the potential antidepressant properties of esnorketamine, we decided to reduce the daily dose in our study to 1.25 mg/kg, to prevent overtreatment and therefore potential unnecessary side effects. Evidence for the idea that a dose of 1.25 mg/kg of oral esketamine is potentially effective is derived from the case report on oral esketamine by Paslakis et al. [40] and from our pilot study (Smith-Apeldoorn SY, Veraart JKE, Ruhé HG, Aan het Rot M, De Boer MK, Schoevers RA. Oral S-ketamine for treating treatment-resistant depression - a case series. In preparation). 

The dosing regimen in our study is fixed at 90 mg per day, based on the weights of the average Dutch man and woman of 84 and 70 kg, respectively [80]. Fixed doses might facilitate easy translation to a clinical setting. The daily dose is given in three administrations a day. With this dosing schedule, high peak blood concentrations can be prevented. This is expected to minimize acute side effects, thereby contributing to patient well-being, continued blinding and applicability. However, there is a risk of not reaching therapeutic blood levels.

Results from a systematic review by Xu et al. [81] suggest that a single administration of very low-doses of ketamine (e.g. 0.1 or 0.3 mg/kg IV) is associated with lower efficacy compared to 0.5 mg/kg IV. It is unclear whether daily administration of multiple low doses for several weeks could achieve comparable efficacy. At present, we do not know whether the defining criterion for the efficacy of ketamine is the peak blood level of (nor)ketamine that elicits changes, the administered dose cumulated per day, or both. Higher sub-anesthetic doses of ketamine can induce psychotomimetic effects. Whether a subjective psychedelic experience potentially has additional therapeutic value, as seen with other hallucinogenic agents [82], requires further investigation [83]. Blood levels of esketamine and esnorketamine and psychotomimetic effects will be determined and considered when analysing the results.

As a final note, we are aware that there is a risk of long-term side effects with repeated (es)ketamine administration. Cognitive impairment, uropathy, hepatobiliary complications, and tolerance are seen in rodent models and ketamine abusers [84–86]. However, ketamine doses used in these studies were substantially higher than in trials with ketamine for TRD or chronic pain [84]. While we will study side effects closely, further research in which daily low doses of ketamine (cf. this study) are directly compared to intermittent use of higher doses will remain necessary.

Results of our RCT are expected to have potentially important implications for the care of patients with TRD. Our data may yield support for the use of oral esketamine, which could fulfill the urgent need for an easily applicable, safe, repeatable, and effective treatment for patients with TRD. Recruitment is on-going. Patient enrolment started in February 2017 and will continue until 128 patients are included.

## Data Availability

Not applicable.

## Abbreviations

AMT: Autobiographical Memory Test
BAI: Beck Anxiety Inventory
BIA: Budget impact analysis
BSS: Beck Scale for Suicide Ideation
CEQ: Credibility/expectancy questionnaire
CGI: Clinical Global Impression
CRO: Clinical Research Office
CYP: Cytochrome P450
DM-TRD: Dutch Measure for quantification of Treatment Resistance in Depression
DSM-5: 5th edition of the Diagnostic and Statistical Manual of Mental Disorders
DSMB: Data Safety and Monitoring Board
DSS: Dissociation Tension Scale
EQ-5D-5 L: EuroQol 5D
FTND: Fagerström Test for Nicotine Dependence
GCPS: Graded Chronic Pain Scale
HDRS_17_: Hamilton Depression Rating Scale
IDS-SR: Inventory of Depressive Symptomatology
ISDI: Iowa Sleep Disturbance Inventory
IV: Intravenous
MDD: Major depressive disorder
MINI: Mini International Neuropsychiatry Interview
NEO-FFI: NEO Five-Factor Inventory
NMDA: N-methyl-D-aspartic acid
QALYs: Quality Adjusted Life Years
QPE: Questionnaire for Psychotic Experiences
RCT: Randomized controlled trial
ROC: Receiver Operating Characteristics
SAFTEE: Systematic Assessment for Treatment Emergent Events
SHAPS: Snaith Hamilton Anhedonia and Pleasure Scale
TRD: Treatment-resistant depression
UMCG: University Medical Center Groningen
ZGV: Zorggebruik Vragenlijst (Health care use questionnaire)
