# Correction to: Oral esketamine for treatment-resistant depression: rationale and design of a randomized controlled trial

**DOI:** 10.1186/s12888-019-2398-7

**Published:** 2020-01-08

**Authors:** Sanne Y. Smith-Apeldoorn, Jolien K. E. Veraart, Jeanine Kamphuis, Antoinette D. I. van Asselt, Daan J. Touw, Marije aan het Rot, Robert A. Schoevers

**Affiliations:** 1 Department of Psychiatry, University of Groningen, University Medical Center Groningen, PO box 30.0001, 9700 RB Groningen, The Netherlands; 2 Department of Psychiatry, PsyQ Haaglanden, Parnassia Psychiatric Institute, The Hague, The Netherlands; 3 Department of Epidemiology, University of Groningen, University Medical Center Groningen, Groningen, The Netherlands; 4 Department of Clinical Pharmacy and Pharmacology, University of Groningen, University Medical Center Groningen, Groningen, The Netherlands; 5 Department of Psychology, University of Groningen, Groningen, The Netherlands

**Correction to: BMC Psychiatry (2019) 19:375**

**https://doi.org/10.1186/s12888-019-2359-1**

After publication of our article [1] we were notified that Fig. 1 was wrongly presented.

**Fig. 1:**
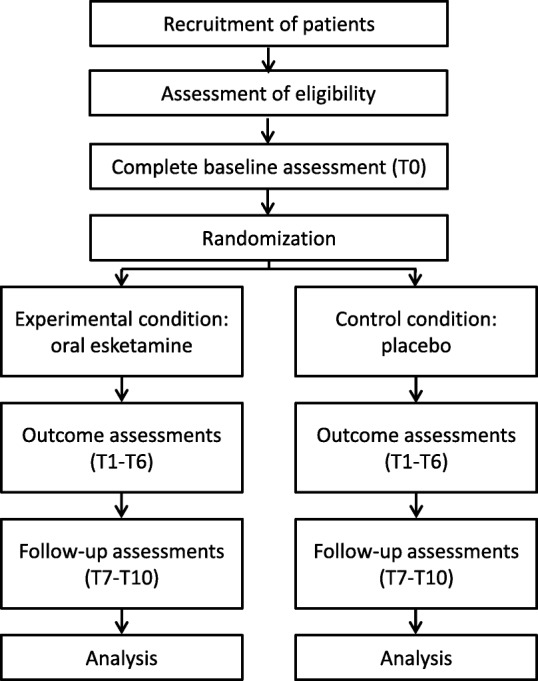
Trial flowchart. Schematic overview of the study design. T: Number illustrates number of weeks after baseline

The correct figure and corresponding title is presented below:

The original article has been corrected.
